# ﻿*Caligusselenecola* sp. nov. (Siphonostomatoida, Caligidae) parasitic on the hairfin lookdown *Selenebrevoortii* (Gill) from the Gulf of California, Mexico, with some comments on *Caritustolii* Rangnekar, 1984

**DOI:** 10.3897/zookeys.1209.120812

**Published:** 2024-08-07

**Authors:** Francisco Neptalí Morales-Serna, Beatriz Yáñez-Rivera, Samuel Gómez

**Affiliations:** 1 Universidad Nacional Autónoma de México, Instituto de Ciencias del Mar y Limnología, Unidad Académica Mazatlán, Joel Montes Camarena s/n, Mazatlán, 82040, Sinaloa, Mexico Universidad Nacional Autónoma de México Mazatlán Mexico

**Keywords:** Biodiversity, Carangidae, Copepoda, Crustacea, parasite, taxonomy

## Abstract

Specimens of a caligid copepod (Siphonostomatoida) were found on the gills of the hairfin lookdown *Selenebrevoortii* (Gill) (Carangidae) from off Mazatlán, Sinaloa (north-western Mexico). This material represents a new species of *Caligus*, *C.selenecola***sp. nov.**, and is assigned to the *diaphanus* species group. Within this group, only *C.kapuhili* Lewis, 1967, *C.laticaudus* Shiino, 1960, *C.macrurus* Heller, 1865, and *C.selenecola***sp. nov.**, have been described with a reduced outer spine 1 on the second exopodal segment of leg 1. These four species can be readily separated by the relative length of the abdomen, and the presence/absence of a process on the myxal area of the female maxilliped, the sternal furca, the postantennal process, and the spiniform process on the basal antennary segment. A full description of the new species is given with some comments on *Caritustolii* Rangnekar, 1984.

## ﻿Introduction

Parasitic copepods of the family Caligidae Burmeister, 1835 (Copepoda: Siphonostomatoida) are commonly found on marine fishes, and are of importance in aquaculture due to the considerable economic losses they can cause to the aquaculture industry (Johnson et al. 2004). Currently, this family includes 29 valid genera (Walter and Boxshall 2024a), of which the most species-rich are *Caligus* Müller, 1785 and *Lepeophtheirus* Nordmann, 1832, with 269 and 123 species, respectively (Walter and Boxshall 2024b, 2024c). On the other hand, several genera contain only a few species. One of these genera is *Caritus* Cressey, 1967 with only two valid species so far, *C.serratus* Cressey, 1967 and *C.tolii* Rangnekar, 1984. Since its discovery, for unknown reasons, *C.tolii* has never been mentioned again: it does not appear in the most relevant and comprehensive literature on the subject (e.g., Dojiri and Ho 2013 and Boxshall and Halsey 2004), and there is no evidence of new records or discussions on it (Geoff Boxshall in litt.), or of any nomenclatural act upon which the species was placed into synonymy or transferred to another genus, or relegated to taxon inquirendum or invalidated. The morphological characteristics of the female of *Caritus* resemble those of *Caligus*, and these two genera have been hypothesized to be closely related (Dojiri and Ho 2013). The major differences between *Caritus* and *Caligus* are the strong reduction of the second and third distal exopodal segments of leg 3, the lamelliform, unarmed endopod of the same leg, and the shape and ornamentation of the exopodal spines of leg 2 (Dojiri and Ho 2013). These three character states are considered herein as probable autapomorphies for *Caritus*. Dojiri and Ho (2013) argued that *Caritus* differs from *Caligus* in the combined lack of the posteriorly directed spiniform process on the basal antennary segment, the postantennal process, and the sternal furca. These structures are present in nearly all the species of *Caligus* and one of these structures may be absent in some species of that genus, but none lacks all these structures (see Dojiri and Ho 2013: 41, table III). They (Dojiri and Ho 2013) purportedly consider the combined lack of these structures as one single character state defining *Caritus*, but the lack of these structures are independent character states. Indeed, the reduction of one or more of these structures seem to have occurred randomly within the *Caligus* group sensu Dojiri and Ho (2013), with a gradual trend towards the reduction of the sternal furca, the postantennal process, and the process on the second antennary segment, and their complete loss in *Caritus*.

Twenty-nine species of *Caligus* and their hosts had been reported from Mexico by 2016 (Morales-Serna et al. 2016: 144–150, app. 1). Since then, four additional, already known, species of *Caligus* have been recorded from Mexican waters: *C.asperimanus* Pearse, 1951 and *C.curtus* Müller, 1785 were found attached to individuals of the spotted rose snapper *Lutjanusguttatus* (Steindachner) (Perciformes: Lutjanidae) from off Michoacan and Guerrero states in south-western Mexico (Villalba-Vasquez et al. 2022); *C.dasyaticus* Rangnekar, 1957 was reported attached to the spotted eagle ray *Aetobatusnarinari* (Euphrasen) (Myliobatiformes: Aetobatidae) and to the southern stingray *Hypanusamericanus* (Hildebrand & Schroeder) (Myliobatiformes: Dasyatidae) from the southern Gulf of Mexico (Rodríguez-Santiago et al. 2016); *C.xystercus* Cressey, 1991 was found attached to the schoolmaster *Lutjanusapodus* (Walbaum) (Eupercaria: Lutjanidae) from the Caribbean (Hernández-Olascoaga et al. 2022). Additionally, another species, *Caligusfajerae* Morales-Serna, Oceguera-Figueroa & Tang, 2017 was described parasitizing the Pacific sierra *Scomberomorussierra* Jordan & Starks (Scombriformes: Scombridae) from off Mazatlán (Sinaloa state, north-western Mexico) (Morales-Serna et al. 2017). Several species have been placed into synonymy since 2016 (e.g., *C.aesopus* Wilson, 1921 and *C.bennetti* Causey, 1953 are currently considered synonyms of *C.lichiae* Brian, 1906 and *C.macrurus* Heller, 1865, respectively), and an updated table of the species of *Caligus* reported from Mexico is presented.

This contribution deals with the description of a new species of *Caligus* found during a recent survey of the metazoan parasites of the marine fish *Selenebrevoortii* (Gill) (Carangidae) from the southeastern Gulf of California. The new species lacks the spinelike process on the basal segment of the antenna, the postantennal process, and the sternal furca, and—most interestingly—spine 1 of the second exopodal segment of the first swimming leg is reduced, and spines 2 and 4 lack the accessory process. The new species is attributable to the *diaphanus* species group of Boxshall (2018), and share the reduced outer spine 1 on the second exopodal segment of leg 1 with *C.kapuhili* Lewis, 1967, *C.laticaudus* Shiino, 1960, and *C.macrurus*. However, these species are readily separated by several characters mentioned below.

## ﻿Materials and methods

A total of 57 individuals of the hairfin lookdown, *Selenebrevoortii* (mean total length = 23.5 cm) caught off Mazatlán, Sinaloa, Mexico, were directly purchased from local fishermen and subjected to a parasitological examination between June and November 2021. Copepod specimens recovered from the gills of the fish were fixed and preserved in 70% ethanol. The specimens were cleared in 85% lactic acid. Drawings were made using a Leica DMLB microscope equipped with a drawing tube.

Abbreviations used through the text, figures, and tables are: **P1–P6**, leg 1–leg 6; **EXP**, exopod; **ENP**, endopod; **EXP (ENP)1 (2, 3)**, first (second, third) segment of the exopod (endopod). Morphological terminology follows Kabata (1979), Boxshall (1990), and Huys and Boxshall (1991). Fish classification and names used herein conform to Froese and Pauly (2023). Nomenclature of the apical elements on the second exopodal segment of the first swimming leg follows Ho and Lin (2004) and Dojiri and Ho (2013).

The type-material was deposited in the Copepoda collection of the Instituto de Ciencias del Mar y Limnología, Unidad Académica Mazatlán (**ICML-EMUCOP**), in Sinaloa, Mexico.

## ﻿Systematics

**Order Siphonostomatoida Thorell, 1859**

**Family Caligidae Burmeister, 1835**

**Genus *Caligus* Müller, 1785**

### ﻿ Caligus selenecola sp. nov.

Taxon classificationAnimaliaSiphonostomatoidaCaligidae

BE7DD5EE-5E3E-5E5F-B2EA-68D37CB432A7

https://zoobank.org/BAD3A0A2-EA0B-47FE-82A6-FF39C115D12B

Figs 1 , 2 , 3 , 4 , 5 , 6 , 7 , 8 , 9 , 10 , 11 , 12

#### Type host.

Hairfin lookdown *Selenebrevoortii* (Gill) (Carangidae).

#### Type locality.

Mexican Pacific, off Mazatlán Port (23°12'N, 106°26'W), Sinaloa, Mexico.

#### Prevalence.

15% (9/57).

#### Type material.

***Holotype***, adult female preserved in ethanol (ICML-EMUCOP-090621-01), collected on 9 June 2021. ***Allotype***, adult male preserved in ethanol (ICML-EMUCOP-081121-01), collected on 8 November 2021. ***Paratypes***, 1 adult female preserved in ethanol (ICML-EMUCOP-090621-02) from the same host individual as the holotype; 1 adult female preserved in ethanol (ICML-EMUCOP-120921-01) from which pair of antennules and P1 a were dissected and mounted onto two slides, and 1 adult female dissected and mounted onto ten slides (ICML-EMUCOP-120921-02), collected on 12 September 2021; and 1 adult female and 1 adult male preserved in ethanol (ICML-EMUCOP-221121-01) from a single host individual collected on 22 November 2021.

#### Site on host.

Gills.

#### Etymology.

The specific name comes from the host genus name *Selene*, and the Latin suffix -*cola*, inhabitor. It is in the nominative singular, gender masculine.

#### Differential diagnosis.

Caligidae. Female: cephalothoracic shield subcircular, with well-developed distinct paired frontal plates, the latter with large ventral lunules. Genital complex nearly as long as wide, slightly shorter than abdomen. Abdomen indistinctly separated from genital complex. Caudal rami twice as long as wide; armed with six setae. Antennule two-segmented; proximal segment with 27 plumose anterior setae; second segment with 13 naked setae and one aesthetasc. Antenna indistinctly four-segmented; second segment without process; without postantennal process; postantennal area with three setule-bearing papillae. Maxilliped with tiny denticle process in myxal area. Sternal furca absent. P1 biramous; P1ENP vestigial; P1EXP two-segmented; P1 EXP2 with three plumose setae on posterior margin, distally with lateral spine 1 minute, elements 2 and 4 spiniform without accessory process, element 3 longest with membranous inner flange. P2 biramous; ENP and EXP tree-segmented; endopodal segments with patch of surface setules anteriorly. P3 biramous; ENP two-segmented; EXP three-segmented. P4 uniramous; EXP three-segmented; outer spines of EXP3 with transverse strip of membrane (modified pecten) close to insertion of spines. Female P5 vestigial, comprised of small lobe with one seta, and larger elongate lobe with three elements. Male: abdomen with two free somites. Antenna three-segmented; middle segment with two corrugated pads and anterior rows of fine striations; distal segment forming long claw with one accessory process and one tiny seta. Maxillule as in female except for dentiform process with blunt distal process. Maxilliped three-segmented; with two conical projections on myxal area of proximal segment. P5 with three setae. P6 represented by two plumose setae.

#### Description.

**Figure 1.**
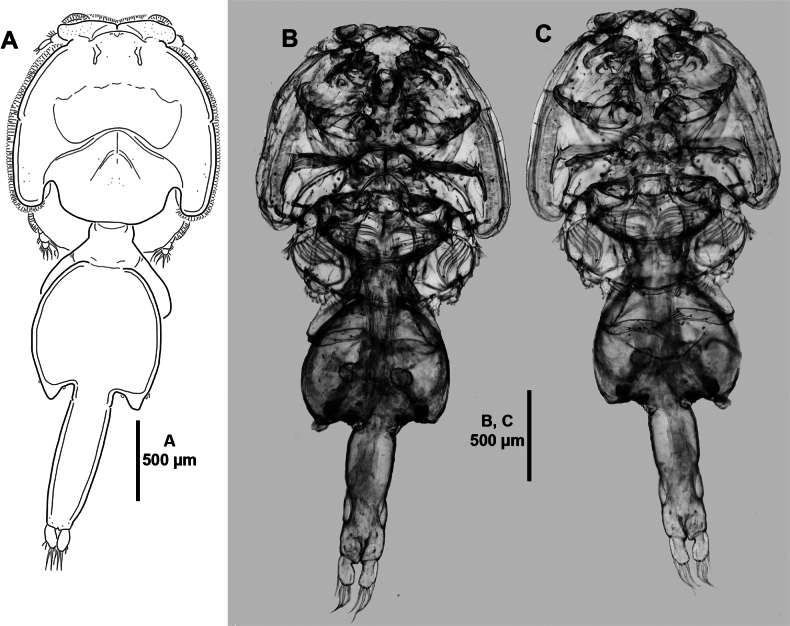
*Caligusselenecola* sp. nov., adult female, holotype **A** habitus, dorsal **B** light microscopy image, habitus, dorsal **C** light microscopy image, habitus, ventral.

**Figure 2.**
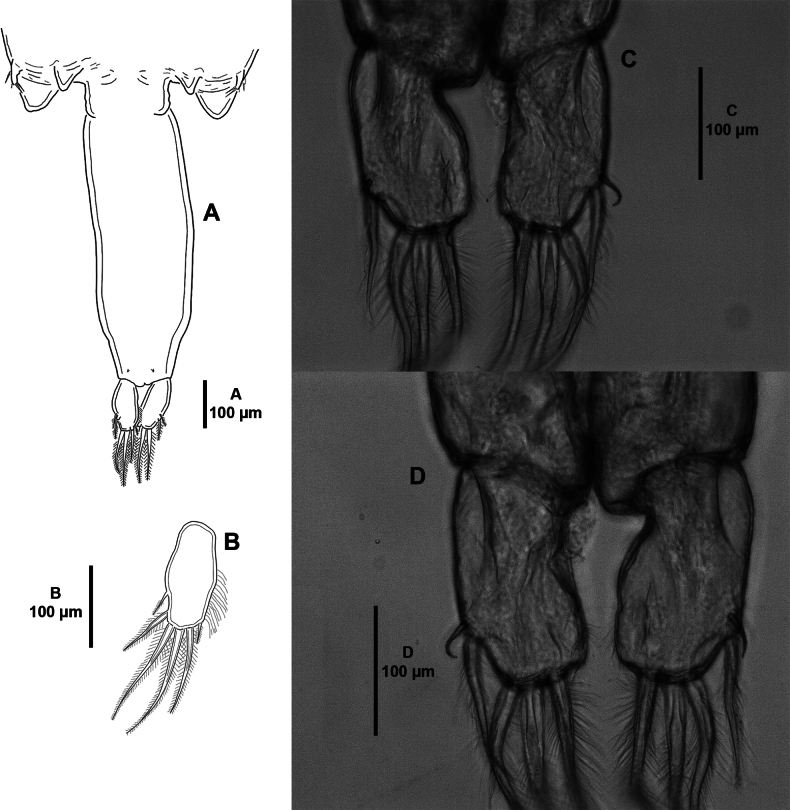
*Caligusselenecola* sp. nov., adult female, holotype **A** posterior region of genital complex, abdomen, and caudal rami, ventral **B** caudal ramus, ventral **C** light microscopy image, caudal ramus, dorsal **D** light microscopy image, caudal ramus, ventral.

**Figure 3.**
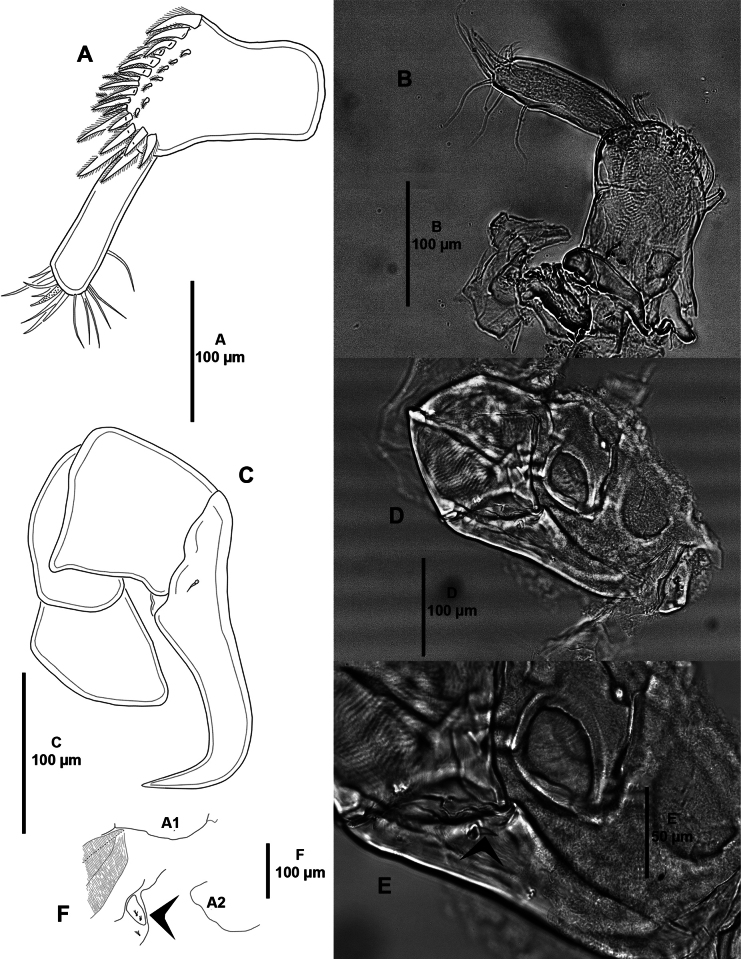
*Caligusselenecola* sp. nov., adult female, holotype (**A, E, F**), paratype ICML-EMUCOP-120921-02 (**B, D, E**) **A** antennule, ventral **B** light microscopy image, antennule, dorsal **C** antenna, ventral **D** light microscopy image, antenna **E** light microscopy image, antenna, showing seta on claw **F** postantennal area showing bases of antennule (a1) and antenna (a2).

**Figure 4.**
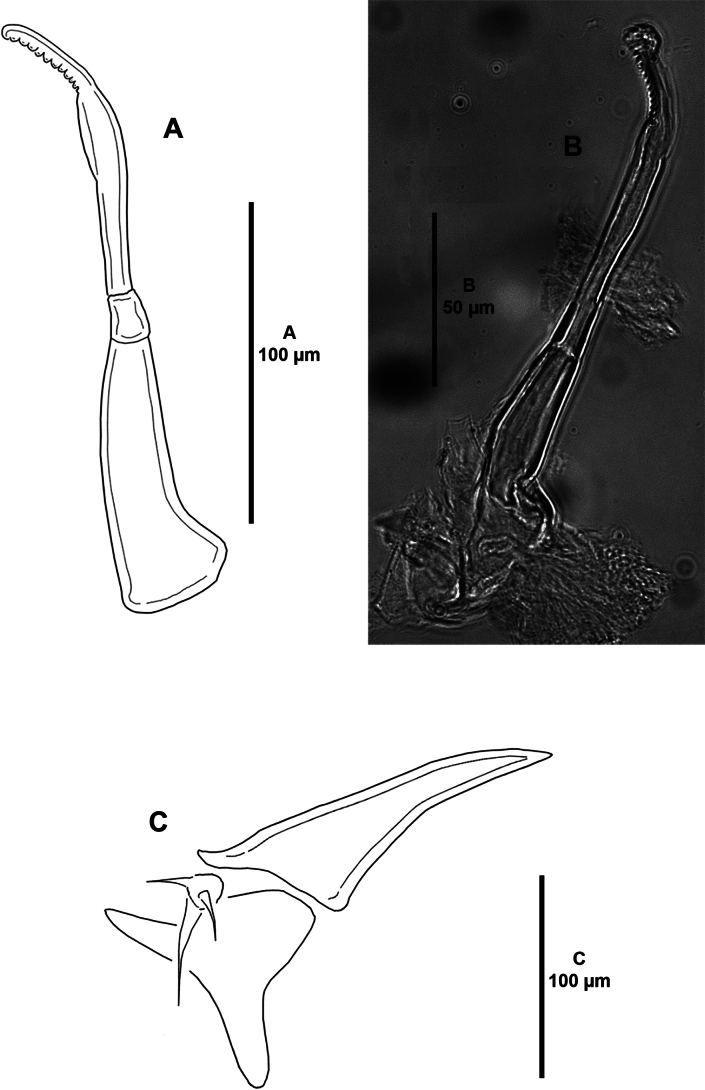
*Caligusselenecola* sp. nov., adult female, paratype ICML-EMUCOP-120921-02 **A** mandible, posterior **B** light microscopy image, mandible **C** maxillule, anterior.

**Figure 5.**
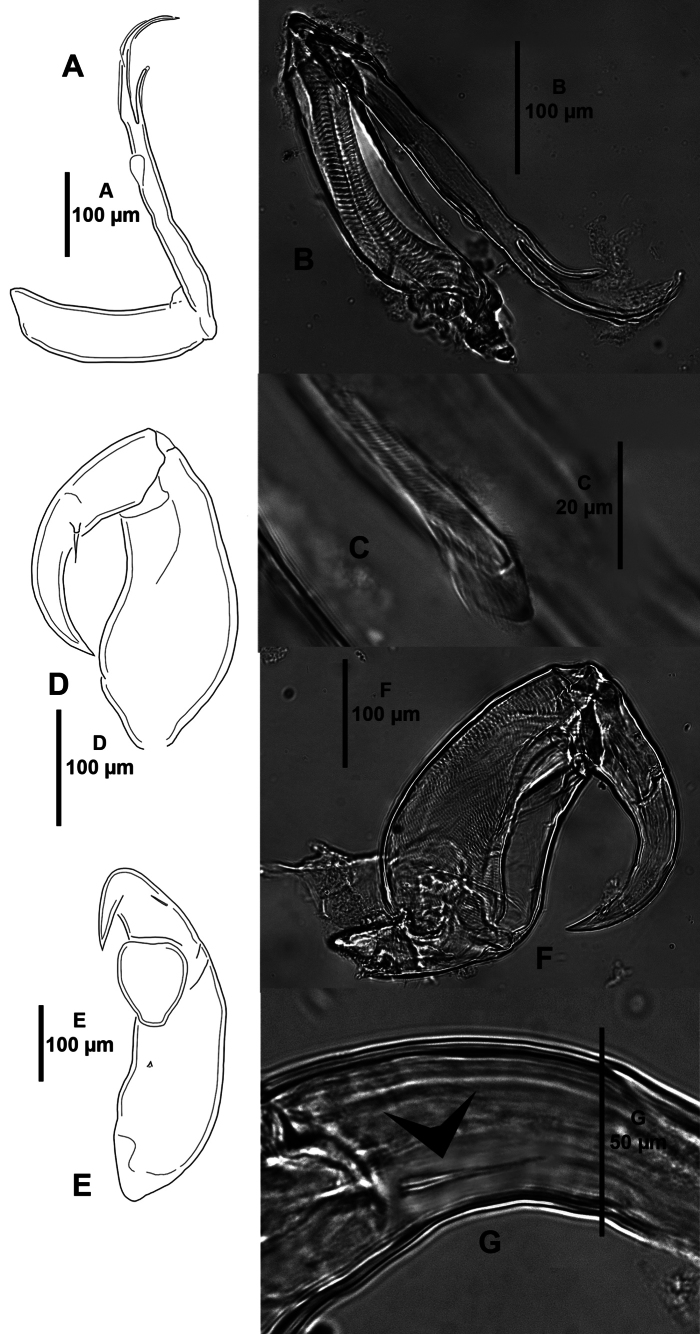
*Caligusselenecola* sp. nov., adult female, paratype ICML-EMUCOP-120921-02 **A** maxilla, anterior **B** light microscopy image, maxilla **C** flabellum of maxilla **D** maxilliped, posterior **E** maxilliped, posteroventral **F** light microscopy image, maxilliped **G** light microscopy image, middle part of claw of maxilliped showing small seta.

**Figure 6.**
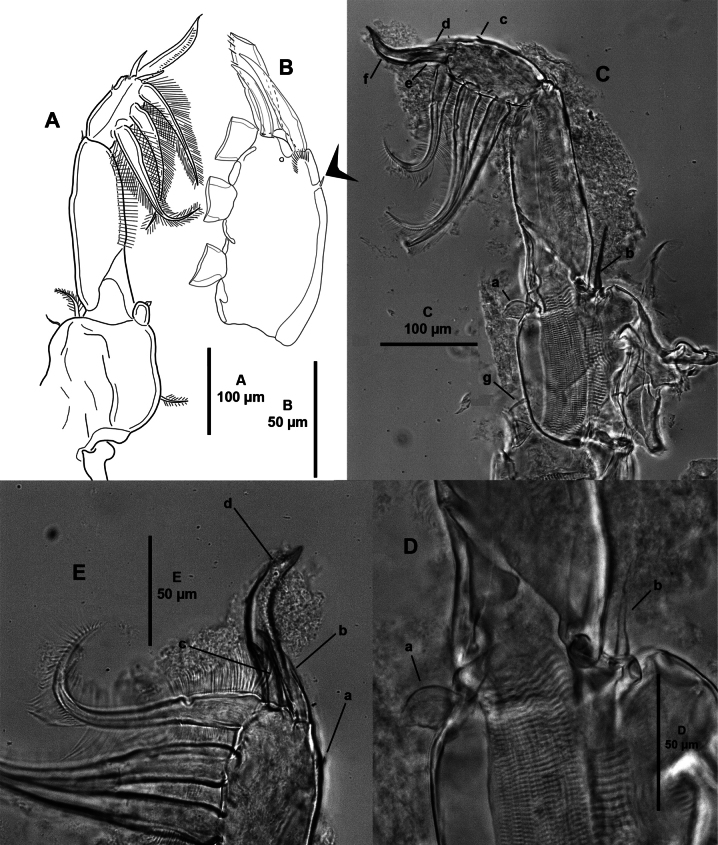
*Caligusselenecola* sp. nov., adult female, paratype ICML-EMUCOP-120921-02 (**A, C–E**), paratype ICML-EMUCOP-120921-01 (**B**) **A**P1, anterior **B** second exopodal segment (spine 1 arrowed), anterior **C** light microscopy image, P1, showing (**a**) endopod (**b**) outer seta of sympod (**c**) spine 1 (**d**) spine 2 (**e**) spine 4 (**f**) seta 3 (**g**) inner seta of sympod **D** light microscopy image, sympod showing (**a**) endopod and (**b**) outer seta **E** light microscopy image, second exopodal segment showing (**a**) spine 1 (**b**) spine 2, (**c**) spine 4, (**d**) seta 3.

**Figure 7.**
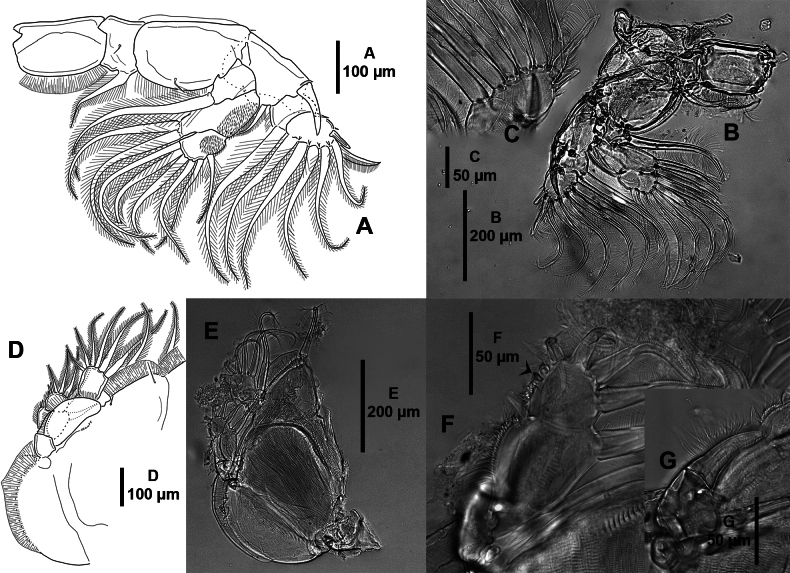
*Caligusselenecola* sp. nov., adult female, paratype ICML-EMUCOP-120921-02 **A**P2, anterior **B** light microscopy image, P2, anterior **C** light microscopy image, third exopodal segment, anterior **D**P3, ventral **E** light microscopy image, P3, anterior **F** light microscopy image, P3EXP showing reduced proximal outer spine **G** light microscopy image, P3 EXP1 showing length of outer spine relative to length of supporting segment.

**Figure 8.**
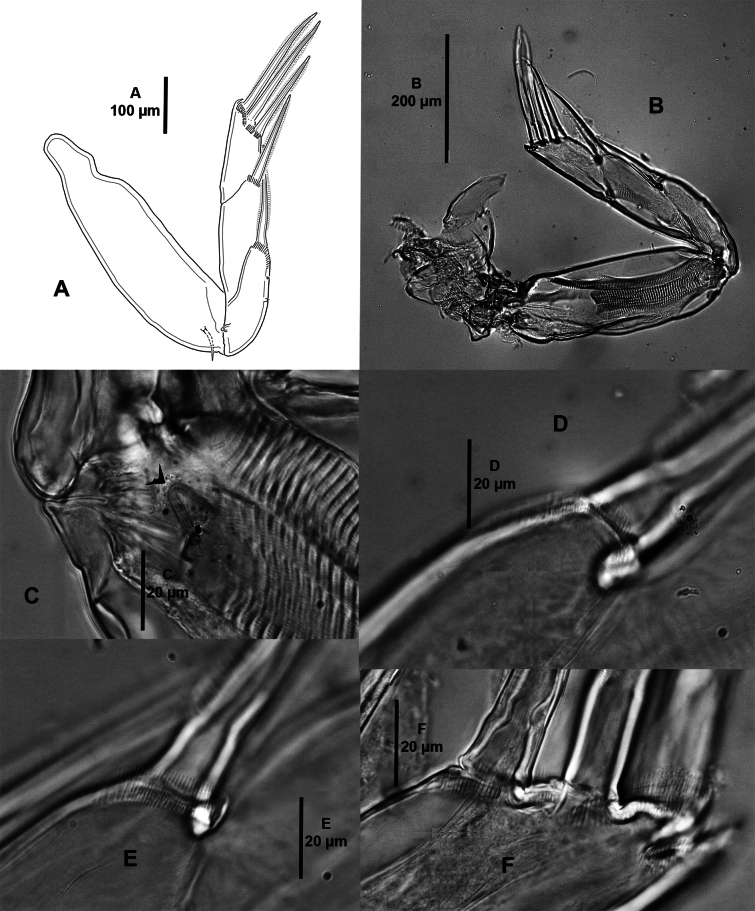
*Caligusselenecola* sp. nov., adult female, paratype ICML-EMUCOP-120921-02 **A**P4, anterior **B** light microscopy image, P4, anterior **C** light microscopy image, protopod of P4 showing seta, anterior **D** outer spine of first exopodal segment showing modified pecten, anterior **E** outer spine of second exopodal segment showing modified pecten, anterior **F** distal spines of third exopodal segment showing modified pectines, anterior.

**Figure 9.**
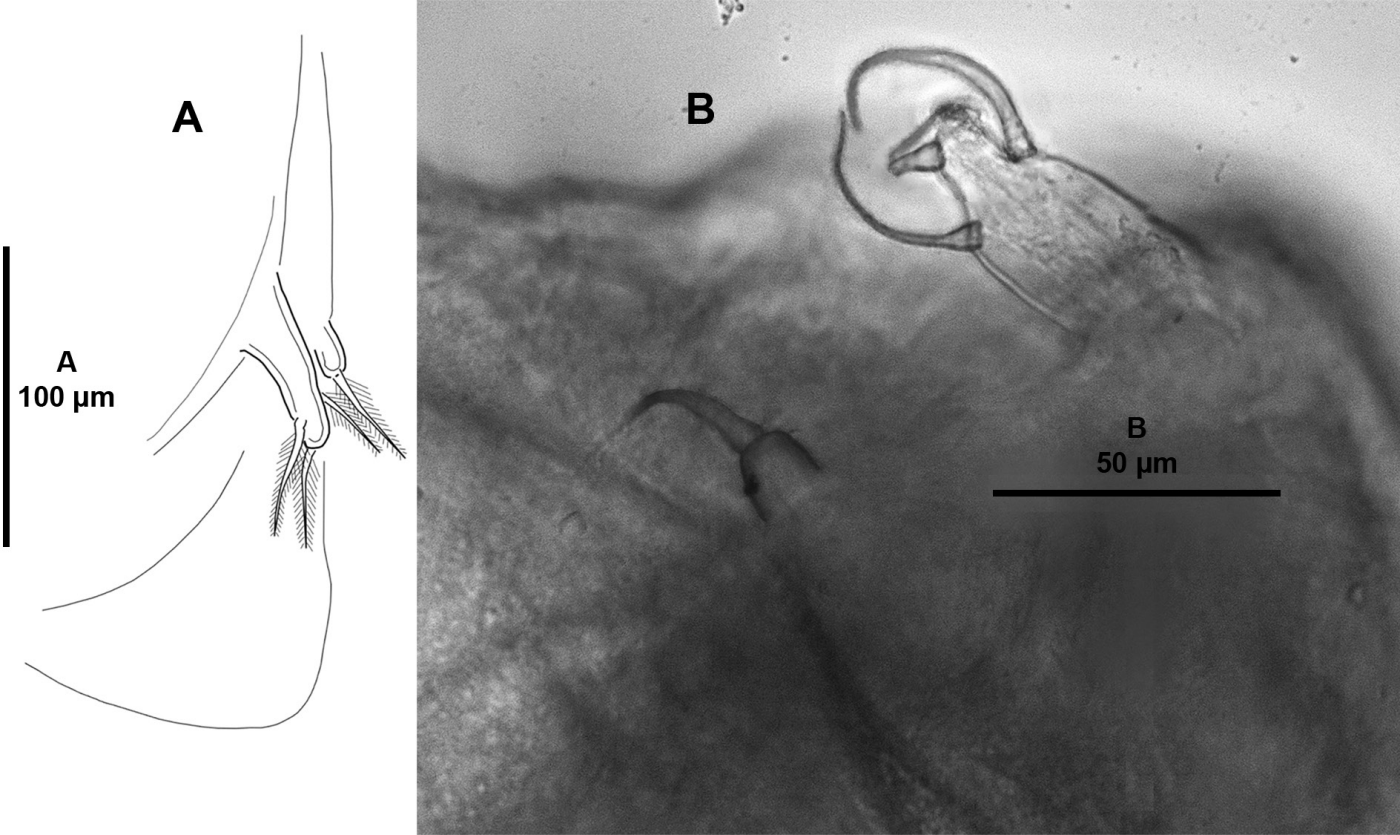
*Caligusselenecola* sp. nov., adult female, holotype (**A**), paratype ICML-EMUCOP-120921-02 (**B**) **A**P5, ventral **B** light microscopy image, P5 ventral.

**Adult female** (Figs 1–9). Mean total body length measured from anterior margin of frontal plate to posterior margin of caudal rami, 3.1 mm (ranging from 2.8 to 3.4 mm; n = 6). Cephalothoracic shield (Fig. 1A–C) subcircular, slightly longer than wide; with well-developed distinct paired frontal plates, the latter with large ventral lunules; with posterior sinuses as shown; medial posterior margin of thoracic zone extending beyond posterolateral margins of cephalothoracic shield; with hyaline membrane along distal margin of frontal plates and laterally. Free fourth pedigerous somite (Fig. 1A–C) slightly wider than long, indistinctly separated from genital complex. Genital complex (Fig. 1A–C) nearly as long as wide, slightly shorter than abdomen, genital complex: cephalothoracic shield length radio 0.7; with posterolateral processes. Abdomen (Figs 1A–C, 2A) indistinctly separated from genital complex; ~ 3.5 × as long as wide; with a slight constriction anteriorly. Caudal rami (Figs 1A–C, 2A–D) twice as long as wide; armed with six setae of which two short and four long plumose, ornamented with inner row of setules. Egg sacs (not figured) uniseriate.

***Antennule*** (Fig. 3A, B) two-segmented. Proximal segment longer than distal, the former with 27 plumose anterior setae. Second segment cylindrical, bearing 13 naked setae and one aesthetasc.

***Antenna*** (Fig. 3C–E) indistinctly four-segmented. First segment unornamented, second segment without process, third segment unornamented, terminal segment a curved claw with one minute seta. Without postantennal process; postantennal area with three setule-bearing papillae (arrowed in Fig. 3F).

***Mandible*** (Fig. 4A, B) of typical stylet-like structure; with twelve marginal teeth.

***Maxillule*** (Fig. 4C) comprising anterior papilla bearing three unequal naked setae; with posterior moderately long dentiform process.

***Maxilla*** (Fig. 5A–C) two-segmented, brachiform, comprising elongate unarmed lacertus and slender brachium, the latter with flabellum (Fig. 5C) slightly above halfway inner margin and with long calamus and shorter canna; calamus with strips of serrated membrane arranged obliquely around surface; canna with bilateral strips of serrated membrane.

***Maxilliped*** (Fig. 5D–G) subchelate; corpus concave proximally, with tiny denticle process in myxal area; claw with naked seta on posterior surface (arrowed in Fig. 5G).

Sternal furca absent.

***P1*** (Fig. 6A–E) biramous, with slender, naked intercoxal sclerite. Sympod with one inner and one outer plumose seta, and one proximolateral seta. Endopod vestigial, represented by unarmed process bearing one tiny apical element. Exopod two-segmented. P1 EXP1 with inner row of setules and one small spine at outer distal corner; P1 EXP2 with three plumose setae on posterior margin, and four distal elements as follows: spine 1 minute, arising on outer lateral margin of segment (arrowed in Fig. 6B, C, E); elements 2 and 4 spiniform, seemingly without accessory process, the former slightly longer than the latter, both visibly shorter than element 3; the latter longest, with membranous inner flange.

***P2*** (Fig. 7A–C) biramous, with subquadrate intercoxal sclerite bearing distal hyaline membrane. Coxa with one inner plumose seta and one anterior sensillum. Basis with one outer small seta, one inner sensillum, and with inner hyaline membrane. Exopod three-segmented; first segment as long as second and third segments combined, with one plumose inner seta, one outer stout spine, and with dorsally flexed membrane along outer margin; second segment smallest, with one inner long plumose seta, one small outer spine, and ornamented with inner row of setules; third segment with five plumose inner setae, and two outer spines of which proximal shortest and one outer subdistal seta, the latter stout and with inner hyaline membrane. Endopod three-segmented; first segment with one inner plumose seta, with patch of surface setules anteriorly; second segment with two inner plumose setae and ornamented with anterior patch of setules; third segment with six plumose setae and ornamented with anterior patch of setules.

***P3*** (Fig. 7D–G) with coxa and basis fused into flattened apron-like sympod, with one small outer plumose seta near insertion of exopod, one inner long plumose seta near intercoxal sclerite, and two widely separated sensilla along posterior margin. Sympod and intercoxal sclerite with extended strips of hyaline membrane along lateral and free posterior margins. Exopod three-segmented; first segment with one apical spine longer than segment and reaching slightly beyond articulation between second and third exopodal segments; second segment with one outer spine and one inner plumose seta, ornamented with outer row of setules; third exopodal segment with three outer spines (proximal outer smallest), and four plumose setae, ornamented with outer setules. Endopod two-segmented; first segment extended laterally for form velum, and armed with long inner plumose seta; second segment with six plumose setae.

***P4*** (Fig. 8A–F) uniramous. Protopodal segment with 1 distal seta. Exopod three-segmented; first and second segments with one outer spine each; third segment with three outer spines, with transverse strip of membrane (modified pecten) close to insertion of spines. All spines subequal in length.

***P5*** (Fig. 9A, B) vestigial, situated on ventral surface, near outer margin of posterolateral lobe of genital complex; comprised of small lobe with one plumose seta, and larger elongate lobe with three plumose setae.

Armature formula of P1–P4 as follows (Roman numerals for spines; Arabic numerals for setae):

**Table:** 

	EXP	ENP
P1	I-0; I,III,3	vestigial
P2	I-1; I-1; II,1,5	0–1; 0–2; 6
P3	I-0; I-1; III,4	0–1; 5
P4	I; I; III	absent

***P6*** possibly represented by pair of protuberances located posteromedial to P5 (Figs 1A–C, 2A).

**Figure 10.**
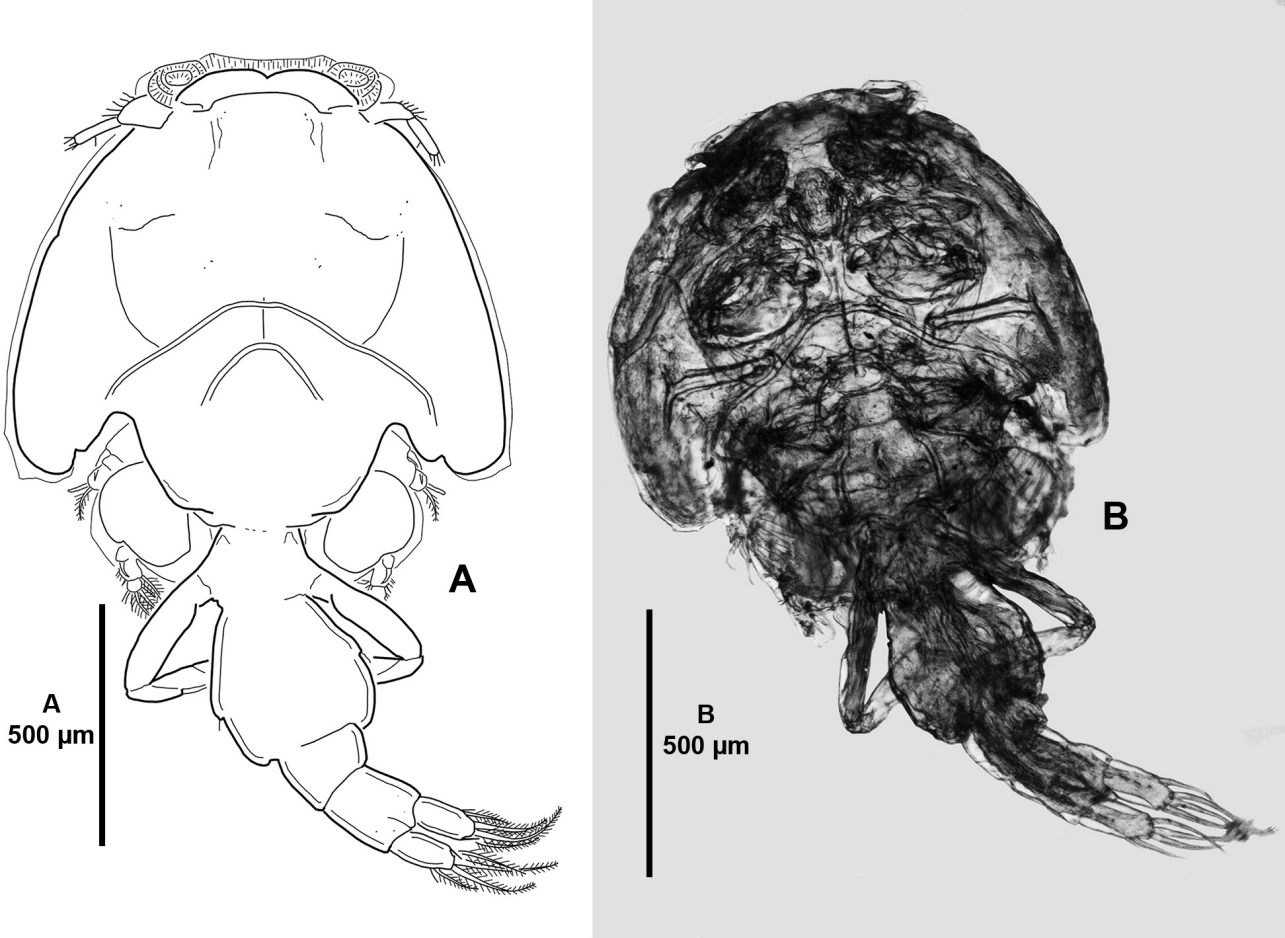
*Caligusselenecola* sp. nov., adult male, allotype **A** habitus, dorsal **B** light microscopy image, habitus, dorsal.

**Figure 11.**
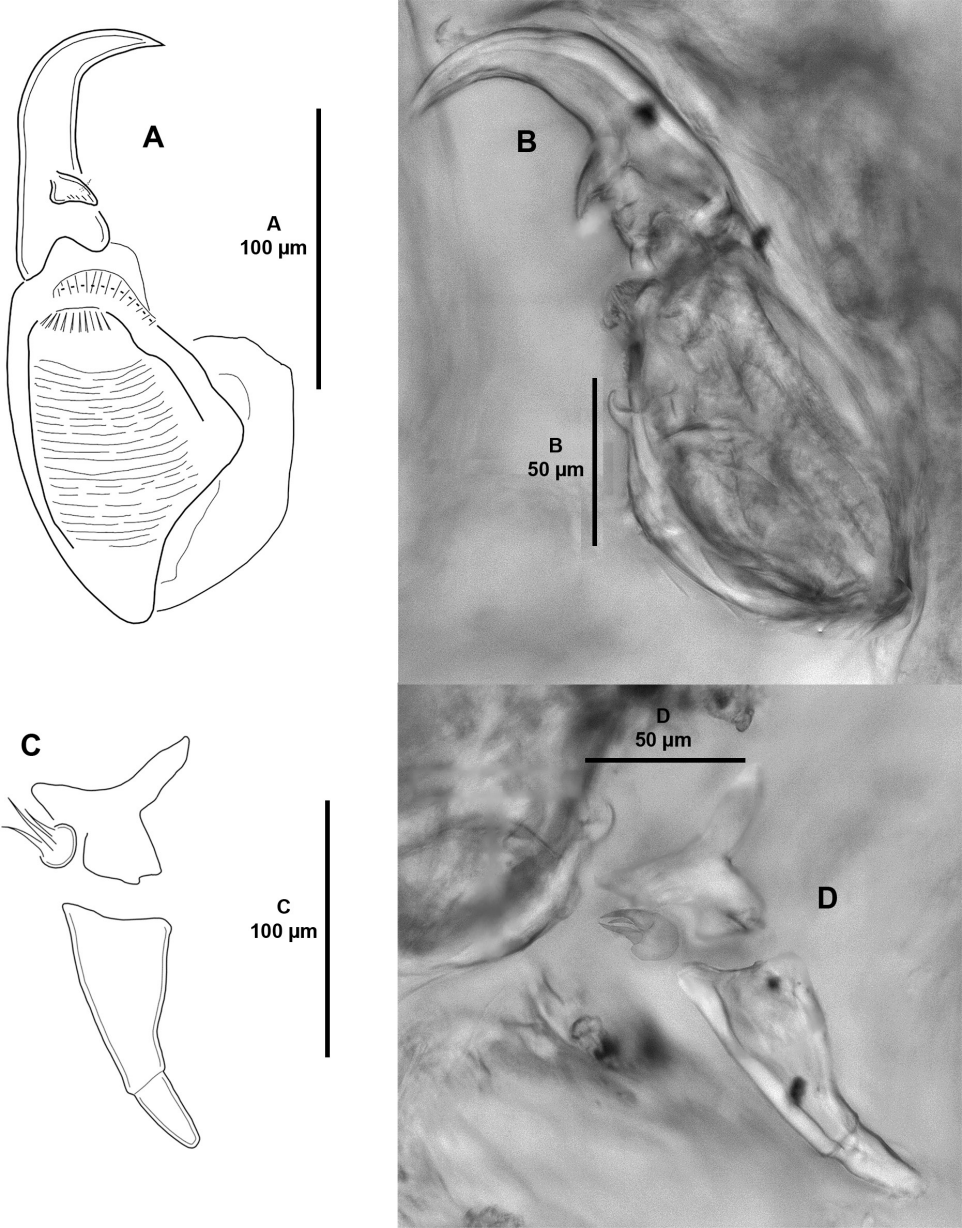
*Caligusselenecola* sp. nov., adult male, allotype **A** antenna, posterior **B** light microscopy image, antenna, anterior **C** maxillule, anterior **D** light microscopy image, maxillule, anterior.

**Figure 12.**
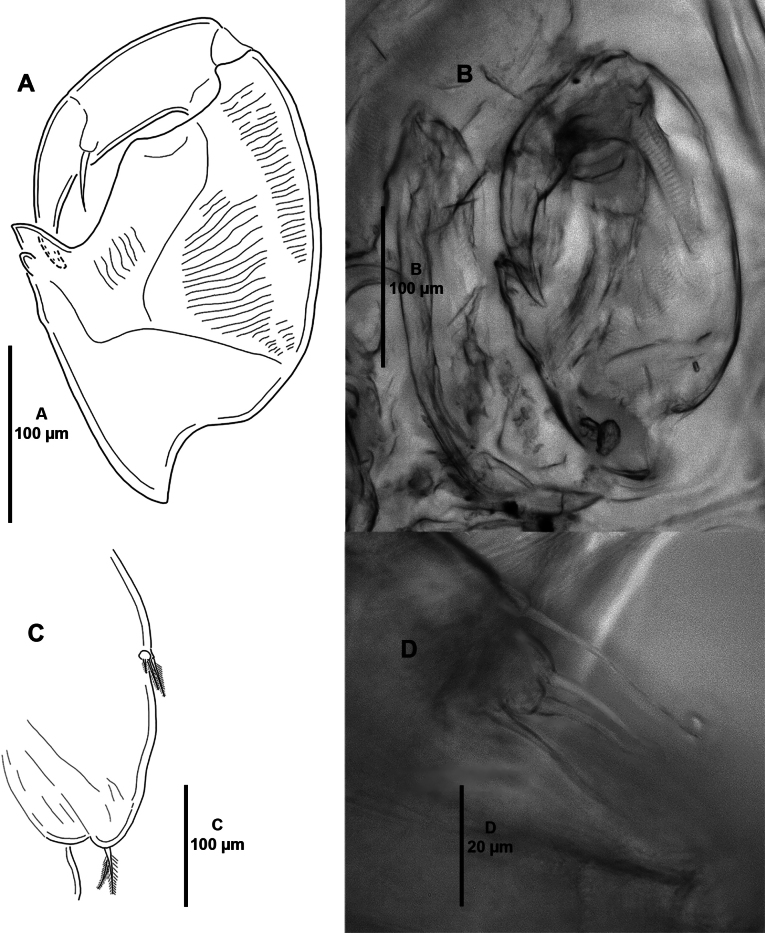
*Caligusselenecola* sp. nov., adult male, allotype **A** maxilliped, posterior **B** light microscopy image, maxilliped, anterior **C** posterolateral margin of genital complex showing P5 and P6**D** light microscopy image, P5, anterior.

**Adult male** (Figs 10–12). Total body length measured from anterior margin of frontal plate to posterior margin of caudal rami 1.9 mm long (*n* = 1). Cephalothoracic shield (Fig. 10A, B) as in female but with narrower anterior region, slightly wider than long. Free fourth pedigerous somite (Fig. 10A, B) slightly wider than long, indistinctly separated from genital complex. Genital complex (Fig. 10A, B) as long as wide. Abdomen (Fig. 10A, B) with two free somites; first somite slightly wider than long, second somite slightly longer than wide. Caudal rami (Fig. 10A, B) ~ 2 × as long as wide, with two short and four long plumose setae. All appendages as in female, except for antenna, maxillule, and maxilliped.

***Antenna*** (Fig. 11A, B) three-segmented; proximal segment small and unarmed; middle segment with two corrugated pads and anterior rows of fine striations; distal segment forming long curved pointed claw with one accessory process and one tiny seta near its base. Postantennal area (not shown) as in female.

***Maxillule*** (Fig. 11C, D) as in female except for dentiform process with blunt distal process.

***Maxilliped*** (Fig. 12A, B) three-segmented; myxal area of proximal segment with two conical projections bearing subterminal tooth; subchela as in female.

***P5*** (Fig. 12C, D) located at approximately midway along lateral margin of genital complex; composed of small lobe bearing three plumose setae.

***P6*** (Fig. 12C) represented by two plumose setae at tip of a posteroventral protuberance on genital complex.

## ﻿Discussion

### ﻿Taxonomic position of *Caritustolii* Rangnekar, 1984

The genus name *Caritus* was coined by Cressey (1967) for *C.serratus* found attached to specimens of the milk fish, *Chanoschanos* (Forsskål) from Nosy Be, Madagascar. Amongst other characters, the genus was diagnosed by the one-segmented abdomen, by the presence of lunules, by the two-segmented P3ENP, and by the lack of dorsal plates, postantennal process and sternal furca. Since the discovery of the type species of the genus, *C.serratus*, in 1967, only one species, *C.tolii* (erroneously synonymized with *C.serratus* in Soler-Jiménez et al. (2019: 95)) found on the inner wall of the operculum of some specimens of the herring, *Tenualosatoli* (Valenciennes) (Dorosomatidae) from Bombay (Rangnekar 1984), had been added. Rangnekar (1984) thought that the one-segmented condition of the abdomen of *C.serratus* observed by Cressey (1967) could be a misinterpretation since he (Cressey 1967: 6, 8; figs 1, 14) showed the female and male abdomen with a slight constriction probably suggesting its two-segmented condition. Rangnekar (1984) wrote that, following Cressey (1967), *C.serratus* lacked the maxillules. This is obviously erroneous (Cressey (1967) clearly described and showed the maxillule of his species) as is Rangnekar’s (1984) written description of this appendage (compare Rangnekar (1984: 345) and his fig. 1 in the same page). More important are the differences in the armature of the P3ENP noticed by Rangnekar (1984), being unarmed in *C.serratus*, but with one seta on P3 ENP1 and six elements in P3 ENP2 in *C.tolii*. Rangnekar (1984: 348) expressed some doubts regarding the phylogenetic importance of the absence of the sternal furca in *Caritus* as a character to justify that genus and believed that Caritus might well be placed as a subgenus of Caligus. Because of the close resemblance between the females of *Caritus* and *Caligus*, these two genera have been hypothesized to be closely related (Dojiri and Ho 2013), being the major differences between them i) the strong reduction of the second and third distal exopodal segment of P3, ii) the lamelliform, unarmed endopod of the same leg, and iii) the shape and ornamentation of the exopodal spines of P2 (Dojiri and Ho 2013), which are regarded here as potential autapomorphies for *Caritus*. That the combined lack of the posteriorly directed spiniform process on the basal antennary segment, the postantennal process, and the sternal furca is one of the major differences between *Caritus* and *Caligus* as argued by Dojiri and Ho (2013) is, in our opinion, not entirely correct. Indeed, these structures are present in nearly all the species of *Caligus*, or one of these structures may be absent in some species, but none lacks all these structures (see Dojiri and Ho (2013: 41, table III)). However, the reduction of one or more of these structures seems to have occurred randomly within the *Caligus* group *sensu*Dojiri and Ho (2013: 402), and a gradual trend towards the reduction of the sternal furca, the postantennal process, and the process on the second antennary segment, seems to be in progress, with their complete loss in *Caritus*. For example, the sternal furca of *Anchicaligusnautili* Stebbing, 1900 —the only species of that genus—is reduced to posteriorly directed sclerotized protrusions, it lacks the postantennal process, and the spinelike projection on the second antennary process is reduced to a rounded protrusion; the sternal furca may be present or absent in the species previously allocated in the former *Sciaenophilus* Beneden, 1852 (= *Caligus* after Özak et al. 2017), and the postantennal process is present, but lacks the posteriorly directed process on the second antennary segment; *Metacaligus* Thomsen, 1949 lacks the sternal furca, and the postantennal process is pointed but reduced in size, and the spinelike projection on the second antennary process is missing in *Metacaligustrichiuri* Krøyer, 1863; *Echetus* Krøyer, 1864, with its type and only species, *E.typicus* Krøyer, 1864, lacks the sternal furca and the process of the postantennal process, and the posteriorly directed process on the second antennary process is reduced in size; *Caligodes* Heller, 1865 possesses the sternal furca and posteriorly directed spinelike process on the second antennary segment, but lacks the postantennal process.

Recent advances showed that i) *C.bennetti* is a junior synonym of *C.macrurus*, and *Sciaenophilus* is a synonym of *Caligus* (Özak et al. 2017), ii) that *Caligodesalatus* Heegaard, 1945 and *Parapetalusspinosus* Byrnes, 1986 belong to *Caligus* but required replacement names, *Caligusseriolicolus* Boxshall, 2018 and *Caligusalepicolus* Boxshall, 2018, respectively (Boxshall 2018), and iii) that *Sinocaligus* Shen, 1957 is synonym of *Caligus* (Boxshall and Barton 2023). Besides regarding some species of *Caligus* as *species inquirendae* and *nomina nuda*, the proposal of several synonymies between some species of *Caligus* and between some species of *Caligus* and *Euryphorus* Milne Edwards, 1840, the reallocation of *Chalimustenuis* Leidy, 1889 to that, currently, invalid genus, and the partial redescription of several poorly-known species, Özak et al. (2017), Boxshall (2018), Boxshall and Bernot (2023), and Boxshall and Barton (2023) contributed importantly to the taxonomy and systematics of *Caligus*. However, being the largest genus within the entire family, and given the inherent complexity of making comparisons to establish new species (Boxshall 2018) it is not clear to what extent the generic diagnosis of *Caligus* has been expanded. The genus could eventually be divided into two or more genera, but a revision of the genus must be conducted first (Dojiri and Ho 2013).

Rangnekar (1984) believed that *C.tolii* was the second species of *Caritus* based on the simultaneous lack of the posteriorly directed spiniform process on the basal antennary segment, the postantennal process, and the sternal furca. The drawings of Rangnekar (1984) are not detailed enough, but *C.tolii* departs from the general scheme of *Caritus* in the shape of the spines of the P2EXP (which look of the normal caligid type), and armature of P3 EXP2–3 (normal, relatively well-developed, and of the caligid type in *C.tolii*, but extremely reduced in *C.serratus*) and P3ENP (well-developed in *C.tolii* but absent in *C.serratus*). Based on the available literature and evidence, it seems that *C.tolii* does not belong to *Caritus*, and is herein proposed to be removed from that genus and reallocated into *Caligus* as *Caligustolii* (Rangnekar, 1984), comb. nov. The original description of the species by Rangnekar (1984) lacks the necessary detail and some characteristics of the *diaphanus* group cannot be verified. Pending its redescription, *C.tolii* comb. nov. is attributed here to the *diaphanus* species group by the combination of i) the armature of the three-segmented P3EXP with I, I, and III spines¸ ii) the presence of three plumose setae on the posterior margin of P1 EXP2, iii) the apparent lack of an accessory process on spines 2 and 3 of P1 EXP2, and iv) lack of the posterior process on the proximal segment of the antenna, and lack of the postantennal process.

### ﻿Taxonomic position of *Caligusselenecola* sp. nov.

Boxshall and Gurney (1980) proposed the *macarovi* group for 28 species. More recently, Boxshall (2018) listed 44 species in that group and, aiming at facilitating the identification process, establishment of new species, and comparison of the species of *Caligus*, he proposed four additional species groups of *Caligus* based on the combination of several morphological character states, the *bonito*, *confusus*, *diaphanus*, and *productus* groups. More recently, Ohtsuka and Boxshall (2019) proposed the *pseudorhombi* species group, and Ohtsuka et al. (2020) grouped some species of *Caligus* in their *undulatus* species group. Of interest here is the *diaphanus* group characterized by i) three-segmented P4EXP with I, I, III spines and ornamented with a modified pecten at the base of each spine, ii) P1 EXP2 with three plumose setae on the posterior margin, iii) spines 2 and 3 on the distal exopodal segment of P1 apparently lacking the accessory processes, iv) P2 ENP2–3 ornamented with surface fine setules, v) outer spines of P2 EXP1–2 aligned close to longitudinal axis of ramus, vi) antenna without posterior process on proximal segment, and vii) tine on post-antennal process vestigial or weakly developed (Boxshall 2018). To this group belong *C.auriolus* Boxshall & Barton, 2023, *C.cybii* Bassett-Smith, 1898c, *C.diaphanus* Nordmann, 1832, *C.fajerae*, *C.kanagurta* Pillai, 1961, *C.kapuhili*, *C.laticaudus*, *C.macrurus*, *C.pagelli* Delamare Deboutteville & Nunes-Ruivo, 1958, *C.pagri* Capart, 1941, *C.pelamydis* Krøyer, 1863, *C.platytarsis* Basset-Smith, 1898a, *C.robustus* Bassett-Smith, 1898b, *C.seriolae* Yamaguti, 1936, *C.stromatei* Krøyer, 1863, *C.tanago* Yamaguti, 1939, *C.tenuis* (Beneden, 1852), *C.tolii* comb. nov., and *C.torpedinis* Heller, 1865 (Boxshall 2018; Boxshall and Barton 2023; Boxshall and Bernot 2023), and Boxshall and Barton (2023) provided a key to the species of this group.

The new species is attributable to the *diaphanus* species group of Boxshall (2018), and following Boxshall and Barton’s (2023) key to the species of that species group, the new species keys out as an intermediate form between *C.torpedinis* (= *C.rotundigenitalis* Yü, 1933 after Boxshall and Bernot (2023)) and *C.pagri*, with the lateral margins of the genital complex slightly convex and with the outer spine of P3 EXP1 reaching slightly beyond the articulation between EXP2 and EXP3. On the other hand, in addition to *C.selenecola* sp. nov., a very small outer spine 1 of P1 EXP2 has been documented for several species of the genus, i.e., *C.balistae* Steenstrup & Lütken, 1861, *C.creyessorum* Kabata, 1992, *C.cookeoli* Ho & Lin, 2010, *C.dactylopteni* Uma Devi & Shyamasundari, 1981, *C.kapuhili*, *C.laticaudus*, *C.macrurus*, *C.nataliae* Boxshall, 2018, *C.praecinctorius* Hayes, Justine & Boxshall, 2012, *C.pseudorhombi* Boxshall, 2018, *C.sclerotinosus* Roubal, Armitage & Rohde, 1983, and *C.seriolae*, and it has not been observed in many others, i.e., *C.calotomi* Shiino, 1954b, *C.diaphanus*, *C.hamatus* Heegaard, 1955 (recently, Boxshall and Bernot (2023: 562) announced the submission of a case to the International Commission of Zoological Nomenclature (ICZN) to propose that *C.undulatus* Shen & Li, 1959 be given precedence over *C.hamatus*, but the case has not yet been resolved), *C.hobsoni* Cressey, 1969, *C.longicaudus* Bassett-Smith, 1898b, *C.robustus*, *C.sensorius* Heegaard, 1962, *C.sepetibensis* Luque & Takemoto, 1996, and *C.suffuscus* Wilson, 1913. Rangnekar (1984) described the P1 EXP2 of *C.tolii* with three plumose setae on the posterior margin, and four well-developed distal elements. The description of an additional outer minute spine on the outer distal corner of P1 EXP2 needs to be verified, but it is probably a pectinate membrane at the base of spine 1 also observed in *C.serratus* (see Dojiri and Ho 2013: 163, fig. 58f), and other caligids.

Within the first group of species above, only *C.cresseyorum*, *C.macrurus*, *C.nataliae*, *C.praecinctorius*, *C.pseudorhombi*, and *C.sclerotinosus* have been observed possessing an accessory process on spines 2 and 3 of P1 EXP2, but it is highly probable that such processes may be evident under electron microscopy (Boxshall 2018), as recently shown for *C.macrurus* (Özak et al. 2017). Also, within that group of species for which a small spine 1 of P1 EXP2 has been unequivocally observed, only a few species besides *C.selenecola* sp. nov., belong to the *diaphanus* species group, i.e., *C.kapuhili*, *C.laticaudus*, and *C.macrurus*. *Caligusselenecola* sp. nov. can be readily separated from the other three species by i) the relative length of the abdomen (extremely elongate, ~ 1.5 × as long as the cephalothorax, P4-bearing somite, and genital complex combined in *C.macrurus*, shorter than genital complex in *C.kapuhili* and *C.laticaudus*, but as long as genital complex in the new species); ii) shape of the female maxilliped (with process on the myxal margin in *C.kapuhili* and *C.laticaudus*, but with smooth myxal margin in *C.macrurus* and in the new species), iii) presence/absence of the sternal furca (present in *C.macrurus*, *C.kapuhili*, and *C.laticaudus*, but absent in *C.selenecola* sp. nov.), iv) presence/absence of postantennal process (present in present in *C.macrurus*, *C.kapuhili*, and *C.laticaudus*, but absent in *C.selenecola* sp. nov.), v) presence/absence of the spiniform process on the basal antennary segment (present in *C.macrurus* and *C.kapuhili*, but absent in *C.laticaudus* and *C.selenecola* sp. nov.).

With the addition of *C.selenecola* sp. nov., there are currently 35 species of *Caligus* parasitizing teleosts and elasmobranchs from Mexican waters (Table 1).

**Table 1.** Updated list of the species of *Caligus* reported from Atlantic (A) and Pacific (P) coastal waters of Mexico.

Species	Host	Locality	References
*C.asperimanus* Pearse, 1951	*Lutjanusguttatus* (Steindachner)	Guerrero and Michoacán (P)	Villalba-Vasquez et al. (2022)
*C.bonito* Wilson, 1905	*Cratinusagassizii* Steindachner, *Lutjanusnovemfasciatus* Gill, & *Sardachiliensis* (Cuvier)	Oaxaca and Sinaloa (P)	Ho and Lin (2004), Morales-Serna et al. (2012)
*C.callaoensis* Durán, 1980	*Cynoscionxanthulus* Jordan & Gilbert	Jalisco (P)	Morales-Serna et al. (2014)
*C.chamelensis* Morales-Serna, Pinacho-Pinacho, Gómez & Pérez-Ponce de León, 2014	*Kyphosuselegans* (Peters)	Jalisco (P)	Morales-Serna et al. (2014)
*C.chelifer* Wilson, 1905	Found in plankton	Tamaulipas (A)	Morales-Serna et al. (2012)
*C.chorinemi* Krøyer, 1863	*Caranxcaninus* Günther	Jalisco (P)	Morales-Serna et al. (2014)
*C.confusus* Pillai, 1961	*Caranxcaballus* Günther and *Caranxcaninus* Günther	Jalisco (P)	Morales-Serna et al. (2014)
*C.constrictus* Heller, 1865	*Caranxcaninus* Günther	Sinaloa (P)	Morales-Serna et al. (2012)
*C.curtus* Müller, 1785	*Lutjanusguttatus* (Steindachner)	Guerrero and Michoacán (P)	Villalba-Vasquez et al. (2022)
*C.dasyaticus* Rangnekar, 1957	*Aetobatusnarinari* (Euphrasen) and *Hypanusamericanus* (Hildebrand & Schroeder)	Campeche and Tabasco (A)	Rodríguez-Santiago et al. (2016)
*C.diaphanus* Nordmann, 1832	*Lutjanusperu* (Nichols & Murphy)	Jalisco (P)	Morales-Serna et al. (2014)
*C.elongatus* Nordmann, 1832	*Sphoeroidesannulatus* (Jenyns)	Sonora (P)	Morales-Serna et al. (2012)
*C.fajerae* Morales-Serna, Oceguera-Figueroa & Tang, 2017	*Scomberomorussierra* Jordan & Starks	Sinaloa (P)	Morales-Serna et al. (2017)
*C.haemulonis* Krøyer, 1863	*Bagremarinus* (Mitchill)	Veracruz (A)	Morales-Serna et al. (2012)
*C.hoplognathi* Yamaguti & Yamasu, 1959	*Caranxcaballus* Günther, *Caranxcaninus* Günther, and *Tylosurus paciﬁcus* (Steindachner)	Jalisco (P)	Morales-Serna et al. (2014)
*C.lalandei* Barnard, 1948	*Seriolalalandi* Valenciennes	Baja California (P)	Morales-Serna et al. (2012)
*C.latigenitalis* Shiino, 1954a	*Caranxcaballus* Günther, *Lutjanusargentiventris* (Peters), *Kyphosuselegans* (Peters), *Tylosurus paciﬁcus* (Steindachner), and *Prionuruspunctatus* Gill	Jalisco (P)	Morales-Serna et al. (2014)
*C.lichiae* Brian, 1906	*Caranxcaballus* Günther and *Caranxcaninus* Günther	Jalisco (P)	Morales-Serna et al. (2014)
*C.longipedis* Bassett-Smith, 1898a	*Caranxlugubris* and *Caranxcaninus* Günther	Colima and Jalisco (P)	Morales-Serna et al. (2012, 2014)
*C.macarovi* Gusev, 1951	*Cololabissaira* (Brevoort)	Unspecified (P)	Morales-Serna et al. (2012)
*C.macrurus* Heller, 1865	*Kyphosussectatrix* (Linnaeus) and *Paralabraxmaculatofasciatus* (Steindachner)	Sinaloa (P) and Veracruz (A)	Morales-Serna et al. (2012)
*C.mutabilis* Wilson, 1905	*Balistes* sp., *Calamusbrachysomus* (Lockington), *Chaetodipteruszonatus* (Girard), *Centropomus* sp., *Epinepheluslabriformis* (Jenyns), *Hoplopagrusguentherii* Gill, *Katsuwonuspelamis* (Linnaeus), *Kyphosuselegans* (Peters), *Lutjanusguttatus* (Steindachner), *Lutjanusperu* (Nichols & Murphy), *Microlepidotusbrevipinnis* (Steindachner), *Menticirrhusundulatus* (Girard), *Mugilcephalus* Linnaeus, *Paralabraxclathratus* (Girard), *Paralabraxmaculatofasciatus* (Steindachner), *Paralabraxnebulifer* (Girard), *Paraseleneorstedii* (Lütken), *Sardachiliensis* (Cuvier), and *Scomberomorussierra* Jordan & Starks	Baja California, Guerrero, Nayarit, Oaxaca, Sinaloa, and Sonora (P)	Morales-Serna et al. (2012, 2014)
*C.omissus* Cressey & Cressey, 1980	*Scomberomorussierra* Jordan & Starks and *Scomberomorusconcolor* (Lockington)	Jalisco (P)	Morales-Serna et al. (2012, 2014)
*C.pelamydis* Krøyer, 1863	*Scomberomoruscavalla* (Cuvier)	Veracruz (A)	Morales-Serna et al. (2012)
*C.productus* Dana, 1849–1852	*Balistespolylepis* Steindachner, *Calamusbrachysomus* (Lockington), *Centropomus* sp., *Coryphaenahippurus* Linnaeus, *Katsuwonuspelamis* (Linnaeus), *Lutjanus* sp., *Paralabraxclathratus* (Girard), *Paralabraxmaculatofasciatus* (Steindachner), *Scomberomorussierra* Jordan & Starks, *Seriolalalandi* Valenciennes, and *Sphyraenaargentea* Girard	Baja California, Guerrero, Nayarit, Oaxaca, Sinaloa, and Sonora (P)	Ho and Lin (2004), Morales-Serna et al. (2012)
*C.robustus* Bassett-Smith, 1898b	*Caranxcaballus* Günther and *Caranxcaninus* Günther	Jalisco (P)	Morales-Serna et al. (2014)
*C.rufimaculatus* Wilson, 1905	Found in plankton	Yucatán (A)	Morales-Serna et al. (2012)
*C.sclerotinosus* Roubal, Armitage & Rohde, 1983	*Lutjanus Colorado* Jordan & Gilbert, *Lutjanusguttatus* (Steindachner), and *Lutjanusperu* (Nichols & Murphy)	Jalisco (P)	Morales-Serna et al. (2014)
*C.selenecola* sp. nov.	*Selenebrevoortii* (Gill)	Sinaloa (P)	Present study
*C.serratus* Shiino, 1965	*Calamusbrachysomus* (Lockington), *Caranxcaballus* Günther, *Caranxcaninus* Günther, *Cynoscionxanthulus* Jordan & Gilbert, *Elopsaffinis* Regan, *Haemulonsteindachneri* (Jordan & Gilbert), *Kyphosuselegans* (Peters), *Lutjanusargentiventris* (Peters), *Microlepidotusbrevipinnis* (Steindachner), *Scomberomorussierra* Jordan & Starks, *Sphoeroidesannulatus* (Jenyns), and *Tylosurus paciﬁcus* (Steindachner)	Jalisco and Sinaloa (P)	Morales-Serna et al. (2012, 2013, 2014)
*C.tenuifurcatus* Wilson, 1937	*Centropomusrobalito* Jordan & Gilbert and *Nematistiuspectoralis* Gill	Jalisco (P)	Morales-Serna et al. (2012, 2014)
*C.trachynoti* Heller, 1865	*Trachinotuscarolinus* (Linnaeus)	Campeche, Quintana Roo and Yucatán (A)	Morales-Serna et al. (2012)
*C.tylosuri* (Rangnekar, 1956)	*Tylosurus paciﬁcus* (Steindachner)	Jalisco (P)	Morales-Serna et al. (2014)
*C.undulatus* Shen & Li, 1959	Found in plankton	Yucatán (A)	Suárez-Morales et al. (2012)
*C.xystercus* Cressey, 1991	*Lutjanusapodus* (Walbaum)	Quintana Roo (A)	Hernández-Olascoaga et al. (2022)

## Supplementary Material

XML Treatment for Caligus selenecola

## ﻿Additional information

### Author ORCIDs

Francisco Neptalí Morales-Serna https://orcid.org/0000-0002-2577-5369

Beatriz Yáñez-Rivera https://orcid.org/0000-0002-3192-2142

Samuel Gómez https://orcid.org/0000-0002-8597-8846

### Data availability

All of the data that support the findings of this study are available in the main text.
